# Kinetic and Interfacial Aspects of Bi(III) Ion Electroreduction in the Presence of Acetonitrile and 2-Thiouracil

**DOI:** 10.3390/molecules31183260

**Published:** 2026-09-14

**Authors:** Alicja Natalia Pawlak, Agnieszka Nosal-Wiercińska, Robert Pietrzak, Sebastian Grzyb, Selehattin Yilmaz

**Affiliations:** 1 Department of Analytical Chemistry, Institute of Chemical Sciences, Faculty of Chemistry, Maria Curie-Skłodowska University, Maria Curie-Sklodowska Sq. 3, 20-031 Lublin, Poland; alicja.pawlak@mail.umcs.pl; 2 Faculty of Chemistry, Adam Mickiewicz University in Poznań, Uniwersytetu Poznańskiego 8, 61-614 Poznań, Poland; pietrob@amu.pl; 3 Warsaw Medical and Technical Academy of Applied Sciences, Bitwy Warszawskiej 1920 r. 18, 02-366 Warsaw, Poland; sebastian.grzyb@wamt.edu.pl; 4 Department of Chemistry-Analytical Chemistry, Faculty of Science, Çanakkale Onsekiz Mart University, Çanakkale 17100, Turkey; seletyilmaz@hotmail.com

**Keywords:** interface-controlled electroreduction, mixed aqueous–organic media, S/N/O-donor organic molecule, cap-pair effect

## Abstract

Both bulk solution properties and the molecular organisation of the electrode/solution interface may influence the kinetics and reversibility of an electrode process. In this study, the effects of acetonitrile (ACN) and 2-thiouracil (2-TU) in an acidic perchlorate medium on the electroreduction of Bi(III) ions were investigated. A cyclically renewable liquid silver amalgam film electrode (R-AgLAFE) was used as the working electrode as an alternative to classical mercury electrodes. Direct current polarography (DC), square-wave voltammetry (SWV), cyclic voltammetry (CV), and differential capacitance measurements were used. Increasing the ACN content from 0_(v/v)_ to 50%_(v/v)_ decreased the current response, D_ox_, and effective kinetic parameters α and k_s,app_ while increasing ΔE_p_, indicating decreased apparent reversibility of the electrode process. The addition of 2-TU caused a pronounced increase in the SWV peak current and a decrease in the difference between the anodic and cathodic peak potentials. In the 50%_(v/v)_ ACN solution, the accelerating effect was weakest at the highest 2-TU concentration. The differential capacitance measurements provided evidence consistent with significant reorganisation of the interfacial layer. Overall, the observed acceleration associated with 2-TU is consistent with adsorption-dependent interfacial effects and the possible participation of labile Bi(III)–2-TU-associated species in the near-electrode region.

## 1. Introduction

Bismuth-based electrodes are a widely used, less toxic alternative to mercury electrodes in electroanalysis. They are used, among other things, to detect trace amounts of metals using stripping techniques [1,2,3]. The development of these electrodes has progressed from traditional bismuth-coated electrodes to more advanced composite and multimetallic systems. Notable examples include Bi layers integrated with carbon nanomaterials or polymers, as well as alloys such as Bi-Sn, Bi-Cu, and Bi-Sn-Sb [3,4]. In addition to analytical applications, the electrode/solution interface has a strong influence on the reduction of Bi(III) and the subsequent formation of the metallic phase, making this system valuable for studying the effect of interfacial organisation on electrode kinetics [5,6]. The electroreduction of Bi(III) depends not only on the mass transport and electrode potential but is also related to the solvation and adsorption of ingredients in the solution, as well as the electrical structure of the electric double layer (EDL) [5,6,7,8]. These factors affect the local concentration of electrically active molecules and the observed dynamic parameters. Therefore, changes in the current density, peak voltage, peak resolution, or dynamic parameters should not be attributed solely to a single catalytic or inhibitory process.

Aqueous–organic mixtures effectively modulate both volumetric and interfacial properties. Acetonitrile (ACN) is a widely available, polar, aprotic cosolvent. It alters the solvent composition and the solvation process in mixed environments [9] and can also influence molecular organisation at the electrode–phase boundary. Most research to date has focused on water–alcohol systems. In this study, ACN was used as a contrasting reference cosolvent rather than as an environmentally friendly alternative. Although ACN exhibits a less beneficial solvent profile compared with some short-chain alcohols [10], it provides a unique chemical environment for investigating the effect of the solvent on the electroreduction of Bi(III). The adsorption of ACN on metallic surfaces, including polycrystalline silver, has been demonstrated by double-layer capacitance measurements and charge–potential relationship measurements [11], suggesting that its influence goes beyond bulk transport. The latest research using the R-AgLAFE method suggests that solvent mixtures and organic additives can significantly modify interfacial and electrochemical properties [12,13,14]. 2-thiouracil (2-TU), a thiopyrimidine with S, N, and O donor atoms, can interact with metal ions [15]. Its electrochemical implications may include adsorption, alteration of the electric double layer (EDL), and its interactions with metal ions around the electrode. Without specific spectroscopic or structural data, however, there is no clear evidence that the observed response can be explained by a specific Bi(III)–2-TU complex.

Although bismuth electrodes and the electroreduction of Bi(III) have been well studied, the combined effect of ACN and the S/N/O-donor molecule adsorbing on the electrode at the renewable silver amalgam phase boundary remains poorly characterised. This study analyses the effects of ACN and 2-TU on the electroreduction of Bi(III) at a cyclically regenerated electrode with a liquid silver amalgam layer (R-AgLAFE). The experimental method used constant-current polarography, square-wave voltammetry, cyclic voltammetry, and differential capacitance measurements, with particularly strong attention paid to distinguishing between effects arising from the bulk medium and those resulting from interfacial adsorption.

## 2. Materials and Methods

The experiments were conducted to enable independent monitoring of the effects of the composition of the mixed aqueous–organic electrolyte and 2-thiouracil on the electrochemical response of Bi(III). The analysis was based mainly on voltammetric and polarographic measurements, supplemented by differential capacitance measurements.

### 2.1. Reagents and Solution Preparation

Solutions containing 0.5 mol·dm^−3^ NaClO_4_ and 0.5 mol·dm^−3^ HClO_4_ were used as supporting electrolytes. All aqueous solutions were prepared using ultrapure water purified with a Millipore Milli-Q system, and analytical grade acetonitrile was used to prepare the mixed aqueous–organic media. The pHs of the investigated solutions were 0.2–0.3. Each solution was prepared separately in an aqueous medium and in aqueous–acetonitrile mixtures containing 0_(v/v)_%, 25_(v/v)_%, or 50%_(v/v)_ ACN, designated A–C, respectively. The concentrations of NaClO_4_ and HClO_4_ were kept constant in all media, while only the H_2_O/ACN ratio was varied. Solutions containing Bi(III) ions were prepared directly by dissolving an appropriately weighed amount of Bi(NO_3_)_3_·5H_2_O (Merck Life Science Sp. z o.o., a subsidiary of Merck KGaA, headquartered in Darmstadt, Germany) in the relevant supporting electrolyte to obtain a final Bi(III) concentration of 1.0 × 10^−3^ mol·dm^−3^.

For measurement series involving increasing concentrations of 2-thiouracil, a 2-TU stock solution prepared in the corresponding supporting electrolyte was used. Appropriate aliquots of the stock solution were transferred to the measurement vessel and diluted with the same supporting electrolyte to a final volume of 10.0 mL, thereby obtaining the required 2-TU concentrations without changing the ACN fraction or the concentrations of the supporting-electrolyte components. The solutions were prepared immediately before measurement, mixed thoroughly, and deoxygenated with nitrogen for at least 10 min.

For the aqueous system (A), a 2-TU stock solution with a concentration of 1.6 × 10^−3^ mol·dm^−3^ was prepared in the aqueous supporting electrolyte. For the ACN-containing systems (B and C), stock solutions containing 8.0 × 10^−4^ mol·dm^−3^ 2-TU were prepared in supporting electrolytes with the same ACN fraction as the corresponding measurement medium. Lower 2-TU concentrations were obtained by appropriate dilution with the corresponding supporting electrolyte, whereas the stock solution itself was used for the highest investigated 2-TU concentration in systems B and C.

### 2.2. Electrochemical System

Electrochemical measurements were performed using a μAutolab Type III potentiostat equipped with an FRA2 module ((EcoChemie B.V., Utrecht, The Netherlands), operated with GPES 4.9 software, and an M165D programmable electrode stand (ANKO LAB Sp. z o.o., Warsaw, Poland). A conventional three-electrode system was used, comprising a cyclically renewable liquid silver amalgam film electrode (R-AgLAFE) as the working electrode, an Ag/AgCl/3 mol·dm^−3^ KCl electrode as the reference electrode, and a platinum wire as the auxiliary electrode. The active surface area of the R-AgLAFE was approximately 0.17 cm^2^, determined geometrically from the dimensions of the amalgam-coated cylindrical wire segment. Because the exposed length and diameter of the amalgam-coated wire segment were fixed by the electrode construction, the geometric working area remained nominally constant after each surface renewal cycle. The reference electrode was placed directly in the investigated solution.

A 10.0 mL portion of the investigated solution was introduced into the measurement vessel and deoxygenated with nitrogen for 10 min before measurement. Measurements were conducted at 25.0 ± 0.1 °C. Before each recording, the R-AgLAFE surface was mechanically renewed: the previously exposed amalgam surface was removed and a fresh amalgam surface was brought into the working position. The electrode design, operating principle, and renewal procedure have been described previously [5,6,12,13]. The same solution was retained between consecutive techniques; before each subsequent measurement, the solution was briefly mixed and re-deoxygenated with nitrogen, while the R-AgLAFE surface was renewed before each recording. For each technique, five independent replicate measurements were carried out under identical experimental conditions. The measurement order was not randomised; the same fixed sequence was used throughout the study to maintain procedural consistency. During routine validation of the R-AgLAFE in the present and previous studies, changes in measurement order were repeatedly checked and no systematic order-dependent differences in the electrochemical response were observed under the applied conditions. After completion of a measurement series for a given base system, the electrode was cleaned with approximately 15% HNO_3_, rinsed with distilled water, and re-amalgamated for approximately 30 min. Surface regeneration was verified by repeating the Bi(III) electroreduction measurement in the aqueous supporting electrolyte and comparing the signal with that obtained before the measurement series. Experiments were first conducted for supporting electrolytes with different ACN contents and then for systems containing increasing 2-TU concentrations, beginning with the lowest ACN content.

### 2.3. Measurement Techniques and Recording Conditions

The Bi(III) electroreduction process was investigated using direct current polarography (DC), square-wave voltammetry (SWV), and cyclic voltammetry (CV). DC measurements were performed with a 2 mV potential step. For SWV measurements, a pulse amplitude of 20 mV, a frequency of 120 Hz, and a potential step of 2 mV were used. CV curves were recorded at scan rates from 5 to 1000 mV·s^−1^, with a 5 mV potential step. DC and SWV curves were used to qualitatively evaluate changes in the electrochemical response, whereas CV curves were used to analyse the cathodic and anodic peak positions and their dependence on scan rate.

Differential capacitance curves were recorded to assess changes occurring in the electrical double layer at the R-AgLAFE/solution interface. Measurements were performed over the frequency range 200–1000 Hz, and the resulting curves were extrapolated to zero frequency. Changes in the positions and shapes of capacitance minima and the appearance of additional maxima were analysed as qualitative evidence of changes in the interfacial response, including possible adsorption/desorption and reorganisation of the EDL. Because differential capacitance is not uniquely diagnostic of adsorption, these features were not assigned to a single molecular process.

### 2.4. Determination of Kinetic Parameters and Data Processing

The kinetic parameters of the Bi(III) ion electroreduction process were determined primarily from cyclic voltammetry curves, with their interpretation supplemented by results from direct current polarography, square-wave voltammetry, and differential capacitance measurements.

Because Bi(III) electroreduction is a multistep process and may also be affected by adsorption, solvation, and interfacial reorganisation, the derived transfer coefficient α was treated as an effective parameter, whereas the heterogeneous kinetic quantity was reported as the apparent heterogeneous electrontransfer rate parameter k_s,app_. Accordingly, k_s,app_ was used as a comparative descriptor of changes between the investigated systems rather than as an elementary standard rate constant for a single electron-transfer step. The value *n* = 3 used in the calculations represents the formal overall stoichiometry of Bi(III) reduction to Bi(0) and should not be interpreted as the number of electrons transferred in a single elementary rate-determining step. Detailed equations and methodological assumptions are provided in the Supplementary Material [16,17,18,19,20,21,22].

## 3. Results and Discussion

### 3.1. Effect of Acetonitrile and 2-Thiouracil on the Double-Layer Properties of the R-AgLAFE/Solution Interface

**Figure 1:**
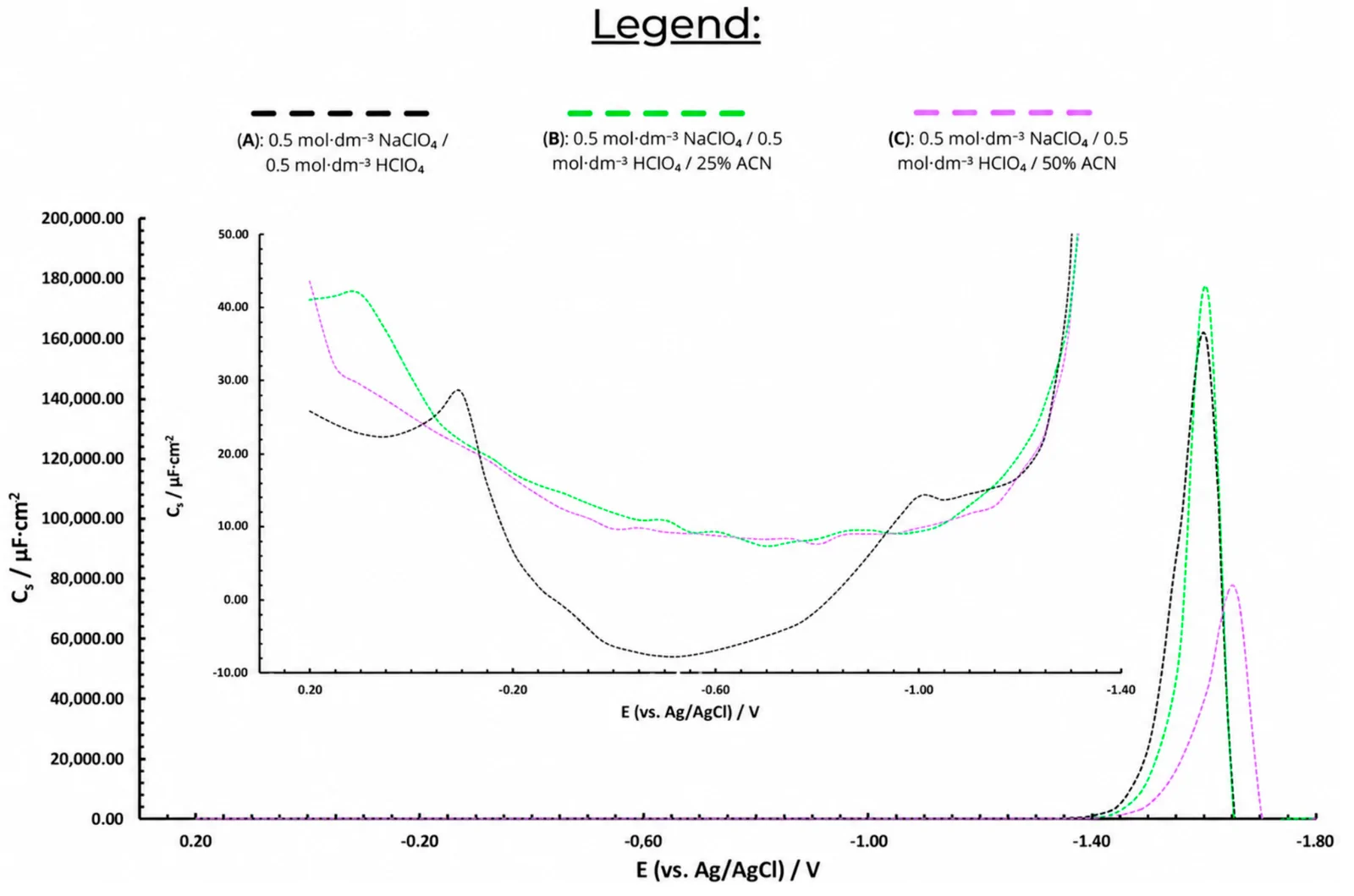
Differential capacitance curves for supporting electrolytes containing 0_(v/v)_, 25_(v/v)_, and 50%_(v/v)_ ACN (A–C).

Differential capacitance curves were used for a qualitative assessment of changes occurring at the R-AgLAFE/solution interface. Two characteristic regions were considered: the region from approximately +0.20 to −1.20 V, covering the capacitance minima and the so-called adsorption region, and the more negative-potential region, extending approximately from −1.25 to −1.80 V, where pronounced capacitance maxima were observed (Figure 1). Changes in the course of the curves, the positions of the minima, and the occurrence of additional maxima provide evidence consistent with modification and reorganisation of the interfacial layer [23,24]. These measurements were therefore interpreted qualitatively and were not used for quantitative determination of adsorption parameters.

#### 3.1.1. Effect of Supporting-Electrolyte Composition

In the aqueous system, the capacitance curve exhibits a broad and pronounced minimum extending from approximately −0.25 to −0.90 V, with the lowest capacitance observed near −0.55 V (Figure 1). The introduction of ACN and increases in its fraction to 25_(v/v)_ and 50%_(v/v)_ clearly shallow this minimum. For solutions B and C, the curves show a similar, relatively flat course over the central potential region, with capacitance values higher than those observed in the aqueous solution. At more negative potentials, capacitance increases again, and the shape of this region depends on the ACN fraction. These observations are consistent with modification of the R-AgLAFE/solution interface by ACN. De Battisti and Trasatti previously demonstrated ACN adsorption at the Hg/aqueous-solution interface using electrocapillary and capacitance measurements [25].

#### 3.1.2. Effect of 2-TU in the Aqueous Medium

**Figure 2:**
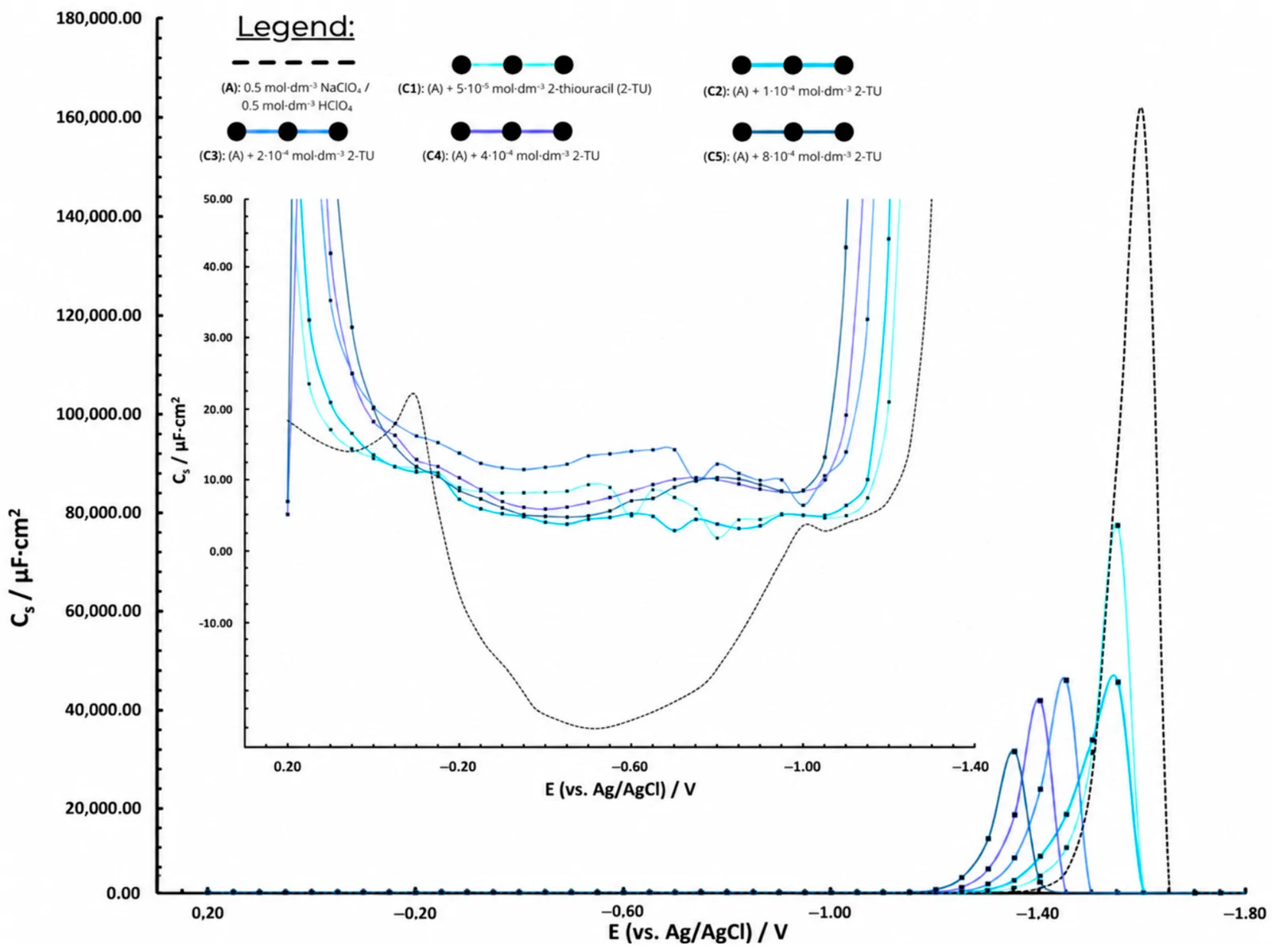
Differential capacitance curves for the aqueous supporting electrolyte (A) in the presence of increasing 2-TU concentrations (indicated in the legend).

After 2-TU was introduced into solution A, the differential capacitance curves clearly changed relative to the supporting electrolyte without the additive. The broad minimum became shallower and the capacitance values increased in the central potential region. At more negative potentials, pronounced capacitance maxima appeared, whose position and intensity depended on the 2-TU concentration (Figure 2). These changes provide evidence consistent with a 2-TU-dependent modification and reorganisation of the R-AgLAFE/solution interface. This interpretation is consistent with earlier studies of thiocarbonyl compounds on mercury. Parsons showed that thiourea adsorption increased the effective thickness of the inner part of the double layer from approximately 4 to 5 Å [26]. For N,N′-dimethylthiourea, the relative surface excess was reported to increase with both adsorbate and NaClO_4_ concentrations [27]. These observations support the sensitivity of EDL properties to thiocarbonyl compounds and electrolyte composition; however, they do not justify assigning individual maxima observed for 2-TU to specific molecular orientations or unambiguous desorption stages.

#### 3.1.3. Effect of 2-TU in Mixed Aqueous–Acetonitrile Supporting-Electrolyte Solutions

**Figure 3:**
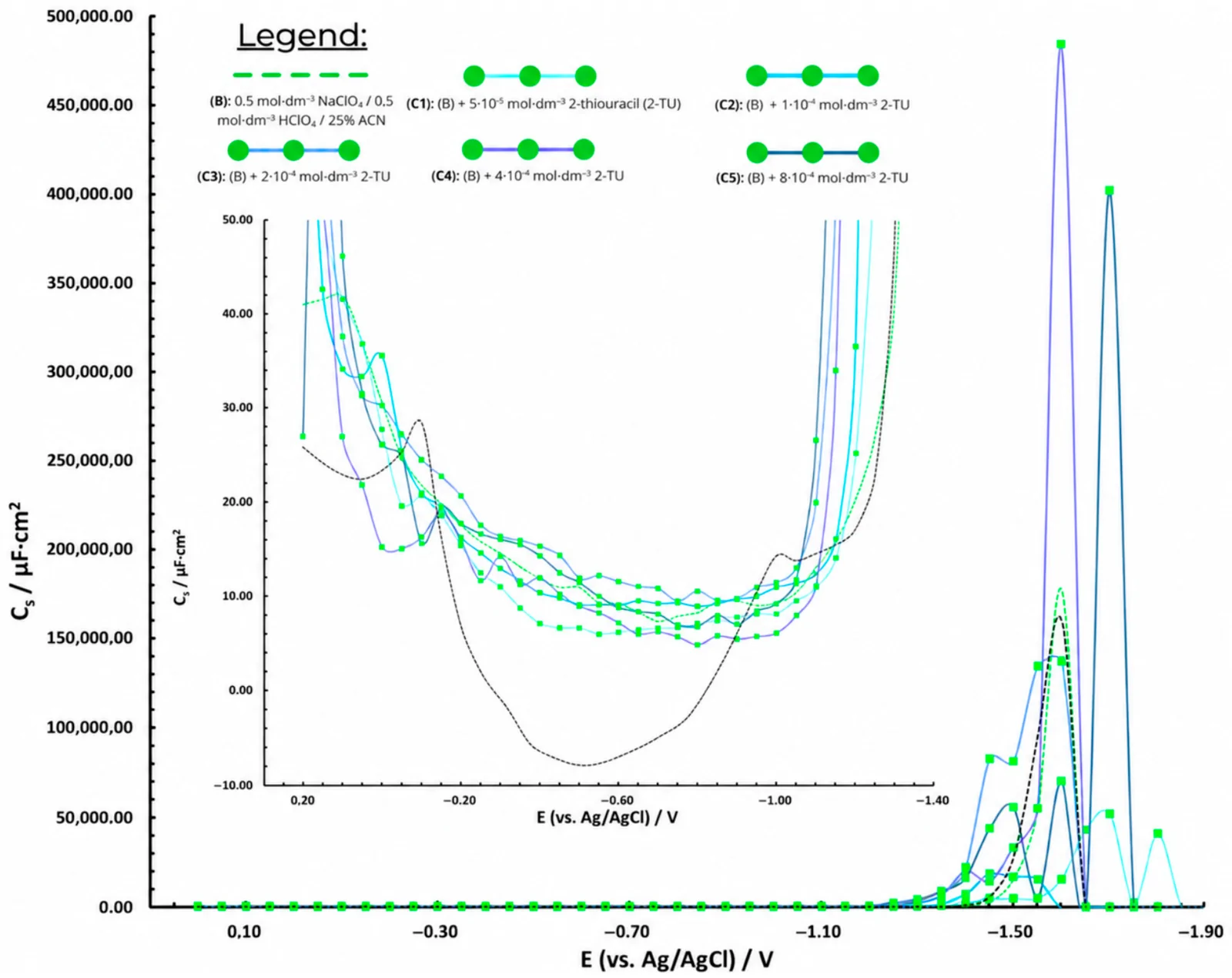
Differential capacitance curves recorded for solution B, containing 25%_(v/v)_ ACN, with increasing 2-TU concentrations (indicated in the legend).

**Figure 4:**
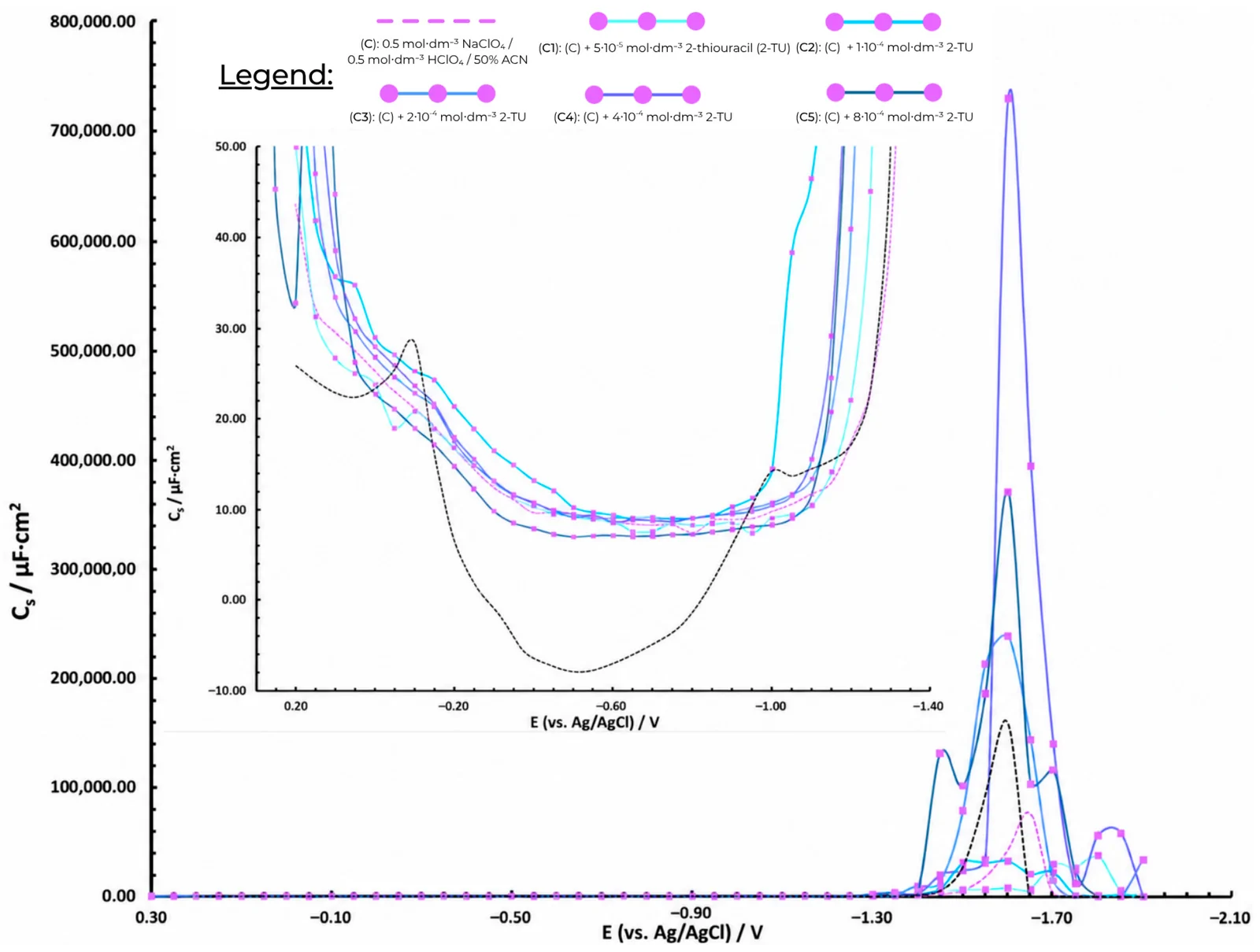
Differential capacitance curves recorded for solution C, containing 50%_(v/v)_ ACN, with increasing 2-TU concentrations (indicated in the legend).

In solutions B and C, containing 25 and 50%_(v/v)_ ACN, respectively, the curves recorded at the individual 2-TU concentrations show broadly similar courses in the central potential region (Figure 3 and Figure 4), with differences mainly in the shallowing of the minima and in local inflections. More pronounced differences appear at more negative potentials, where multiple capacitance maxima are observed. Increasing the ACN fraction is accompanied by changes in the position and separation of these features. This behaviour is consistent with potential- and solvent-dependent reorganisation or partial desorption of a 2-TU-containing interfacial layer and with the known solvent dependence of 2-TU adsorption states [28].

According to the literature, a multipeak differential capacitance response may arise from overlapping contributions to the interfacial response, including solvent reorganisation, changes in dipole orientation, ion accumulation, and adsorption pseudocapacitance [23,24]. For hexanol adsorption on mercury, Darowicki and co-workers demonstrated the joint contribution of double-layer capacitance and adsorption pseudocapacitance [23], whereas Zhen and co-workers related capacitance maxima to potential-dependent water orientation and changes in the electrical double-layer structure [24].

One possible explanation is that different forms or surface states of 2-TU may contribute to the observed interfacial response. Calculations for the gas phase, water, and ACN indicate a clear predominance of the thione form, while more recent kinetic studies suggest that higher-energy thiol forms readily convert into more stable thione forms [29,30]. A small contribution from other tautomers cannot be excluded, particularly in the heterogeneous interfacial environment, although different surface states of the dominant 2-TU form, differing in orientation, degree of ordering, or local H_2_O/ACN environment, appear more plausible. Metal-ion-containing forms may also contribute: adsorption of a Cu(II)–2-TU chelate at a hanging mercury drop electrode has been reported [31], and a review of 2-TU complexes shows that coordination depends on the metal and environmental conditions [15]. A plausible interpretation of the present data is that they were mainly the result of solvent effects, different surface states of 2-TU, possible interactions with Bi(III), and stepwise reorganisation/desorption of the interfacial layer overlap.

The pronounced changes in differential capacitance observed upon addition of 2-TU are consistent with potential-dependent adsorption and reorganisation of the electrode/solution interface. Direct studies of 2-thiouracil at mercury electrodes have shown that several adsorption states may occur depending on the potential, concentration, and temperature. In particular, Wandlowski identified a dilute state of predominantly flat-oriented 2-TU molecules interacting with positively charged mercury through the sulfur-containing moiety, as well as a more compact state exhibiting the thermodynamic characteristics of a two-dimensional condensed film [32]. The organisation of such layers is also strongly solvent-dependent: at the mercury/acetonitrile interface, condensed 2-TU layers were observed. At the same time, the presence of water markedly affected the structure and stability of the ordered film [28]. However, adsorption behaviour cannot be transferred directly between electrode materials. On Ag(111), thiourea electroadsorption/electrodesorption was accompanied by the formation of ordered surface arrangements observed by in situ STM [33]. In contrast, studies on Bi(111) showed substantially weaker and more heterogeneous adsorption, with stable adlayers detectable only under restricted potential and concentration conditions [34]. These observations support the interpretation that the capacitance changes recorded at R-AgLAFE may reflect potential- and solvent-dependent organisation of a 2-TU-containing interfacial layer. Nevertheless, the present electrochemical data do not demonstrate the formation of a two-dimensional condensed phase or establish a specific chemisorption geometry of 2-TU at the R-AgLAFE surface.

### 3.2. Effect of ACN and 2-TU on the Kinetics of Bi(III) Ion Electroreduction

#### 3.2.1. Effect of Solvent Composition

**Figure 5:**
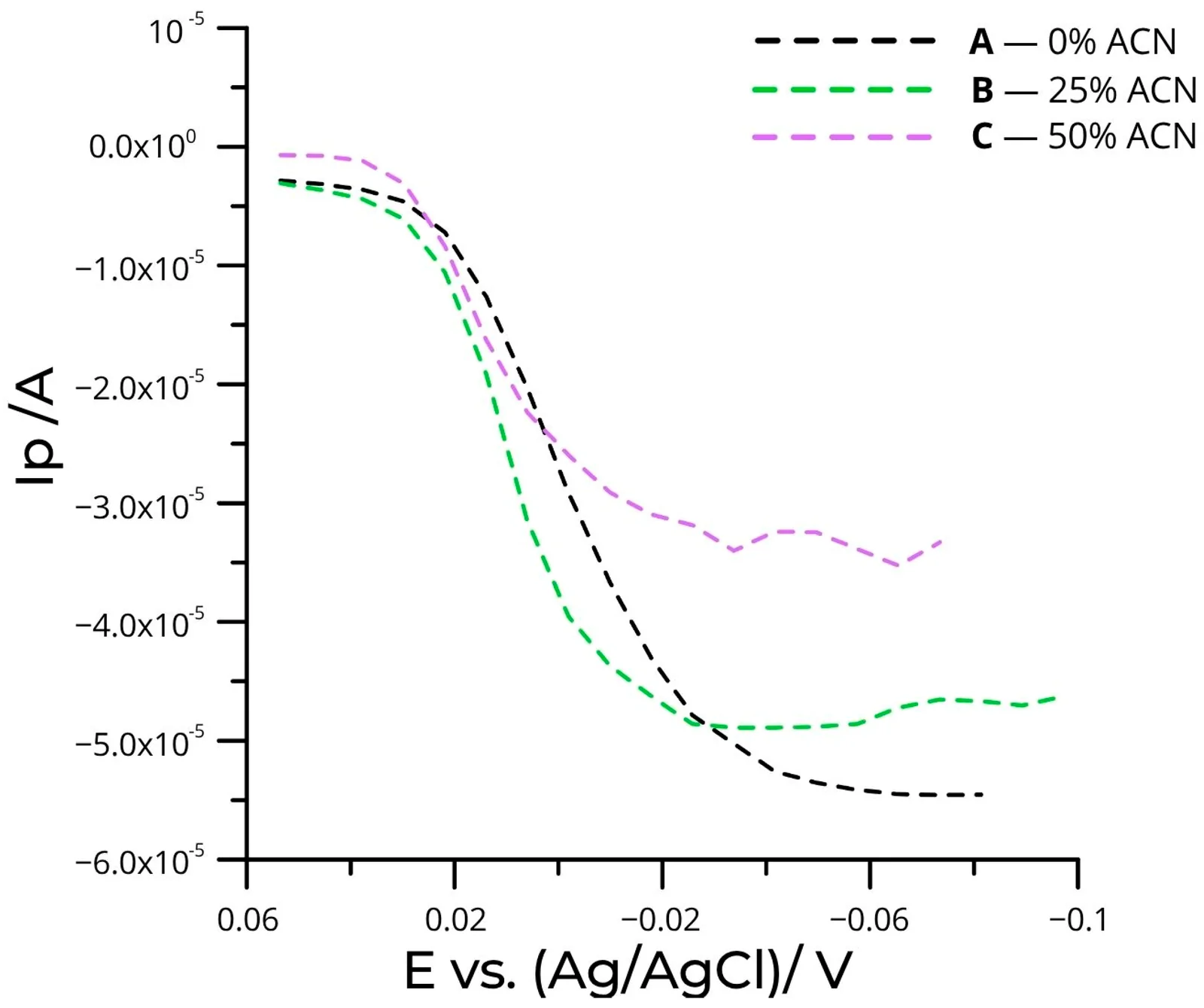
DC polarographic waves of Bi(III) electroreduction in supporting-electrolyte solutions containing different ACN fractions: A, 0% ACN (black dashed line); B, 25%_(v/v)_ ACN (green dashed line); and C, 50%_(v/v)_ ACN (violet dashed line). Supporting electrolyte: 0.5 mol·dm^−3^ NaClO_4_ + 0.5 mol·dm^−3^ HClO_4_; c(Bi(III)) = 1.0 × 10^−3^ mol·dm^−3^.

The effect of the composition of the perchlorate/acetonitrile medium on Bi(III) electroreduction was investigated using DC, SWV, and CV. As the ACN concentration increased, the DC waves became less well defined within the investigated potential range (Figure 5), with the effect most pronounced for solution C. This trend, together with the CV response, indicates decreasing apparent reversibility with increasing ACN fraction. The polarographic wave height also decreased, particularly in solution C. This behaviour may reflect combined transport, solvation, and interfacial effects. The apparent D_ox_ values decreased from 1.71 × 10^−5^ to 7.74 × 10^−6^ and then to 6.02 × 10^−6^ cm^2^·s^−1^ for solutions A, B, and C, respectively. In this multistep system, these D_ox_ values are used as comparative parameters and should not be interpreted as evidence of a purely molecular diffusion effect.

Changes in the electrochemical response with increasing ACN content were also observed by SWV. The absolute Bi(III) peak current decreased from 112.23 μA in solution A to 97.75 μA in solution B and 90.18 μA in solution C. The corresponding SWV curves are provided in Figure S4. Because both transport and interfacial effects may contribute in mixed aqueous–organic media, this decrease was not interpreted as evidence of a single inhibitory mechanism.

**Figure 6:**
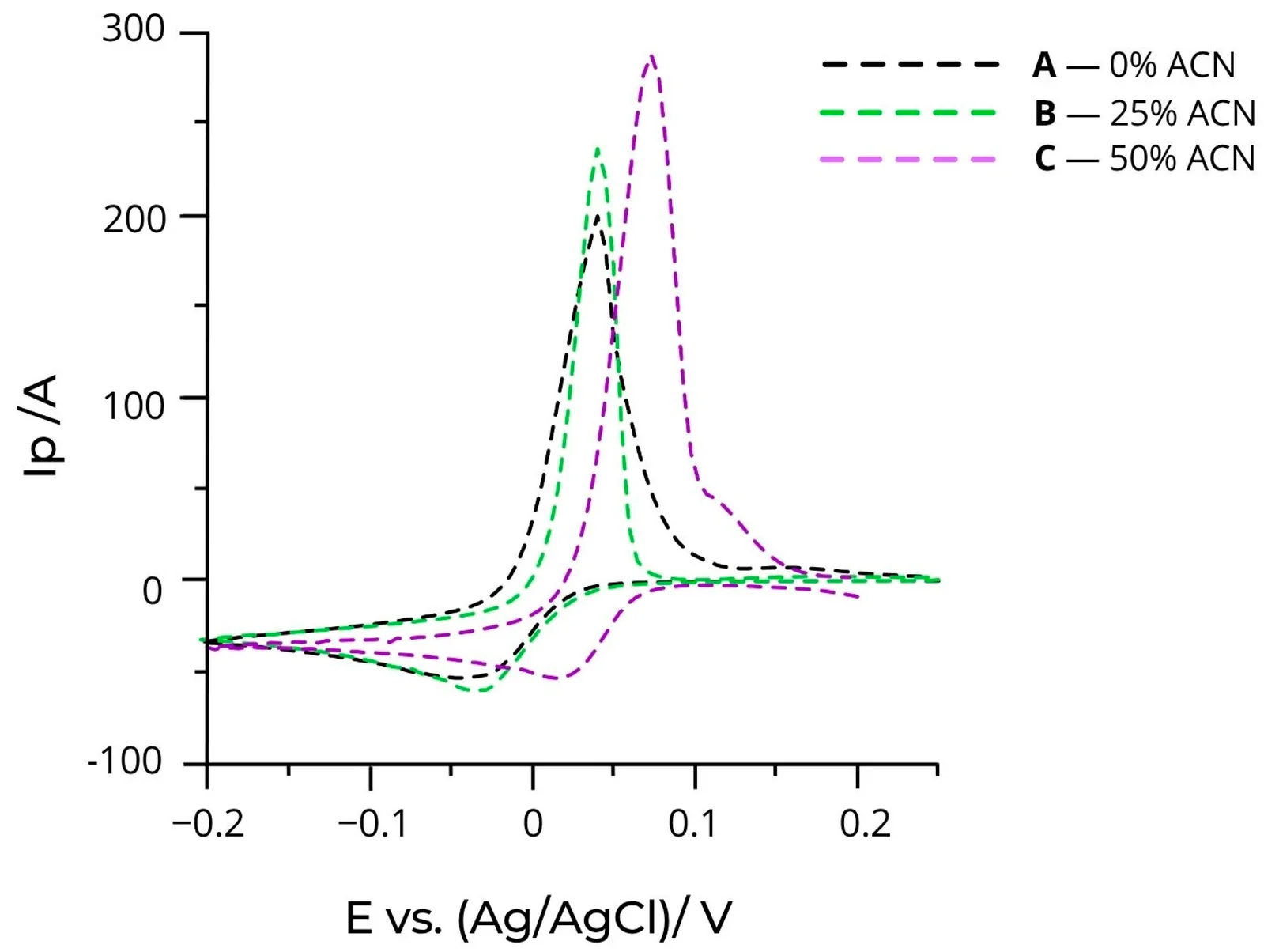
Cyclic voltammetry curves of Bi(III) electroreduction in solutions containing A, 0%_(v/v)_; B, 25%_(v/v)_; and C, 50%_(v/v)_ ACN, recorded at a scan rate of 0.02 V·s^−1^.

With increasing ACN content, the effective cathodic transfer coefficient decreased from 0.33 in solution A to 0.28 and 0.25 in solutions B and C, respectively. The apparent heterogeneous electron transfer rate parameter, k_s,app,_ decreased from 1.66 × 10^−4^ cm·s^−1^ in solution A to 1.41 × 10^−4^ cm·s^−1^ in solution B and 1.14 × 10^−4^ cm·s^−1^ in solution C (Table 1). The same trend is supported by CV measurements (Figure 6). At a scan rate of 0.02 V·s^−1^, ΔE_p_ increased from 84 mV in solution A to 86 mV in solution B and 91 mV in solution C. Taken together, these changes indicate decreasing apparent reversibility with increasing ACN fraction.

The obtained relationships should not be interpreted solely in terms of bulk transport. Bi(III) electroreduction is a multistep process that may also depend on hydration/dehydration and on the organisation of the near-electrode region. ACN adsorption at the mercury/aqueous-solution interface has previously been demonstrated by electrocapillary and capacitance methods [25], supporting the possibility that ACN affects both bulk medium properties and the interfacial response. However, the contributions of these effects cannot be separated on the basis of the present electrochemical data alone. In addition, because the Ag/AgCl/3 mol·dm^−3^ KCl reference electrode was used directly in media with different H_2_O/ACN compositions, a contribution from changes in liquid junction and effective reference potentials cannot be completely excluded; absolute potential shifts between different solvent compositions should therefore be interpreted with caution [35]. Overall, increasing ACN content is associated with a decrease in the apparent electroreduction kinetics and current response of Bi(III), as reflected by decreases in D_ox_, the effective α, and k_s,app_, together with an increase in ΔE_p_. These changes may arise from combined transport, solvation, interfacial, and reference potential contributions rather than from a single inhibitory mechanism.

#### 3.2.2. Effect of 2-TU on Bi(III) Ion Electroreduction in Mixed Aqueous/Acetonitrile Supporting-Electrolyte Solutions

The effect of 2-thiouracil (2-TU) on the Bi(III) ion electroreduction process was investigated in solutions A–C.

**Figure 7:**
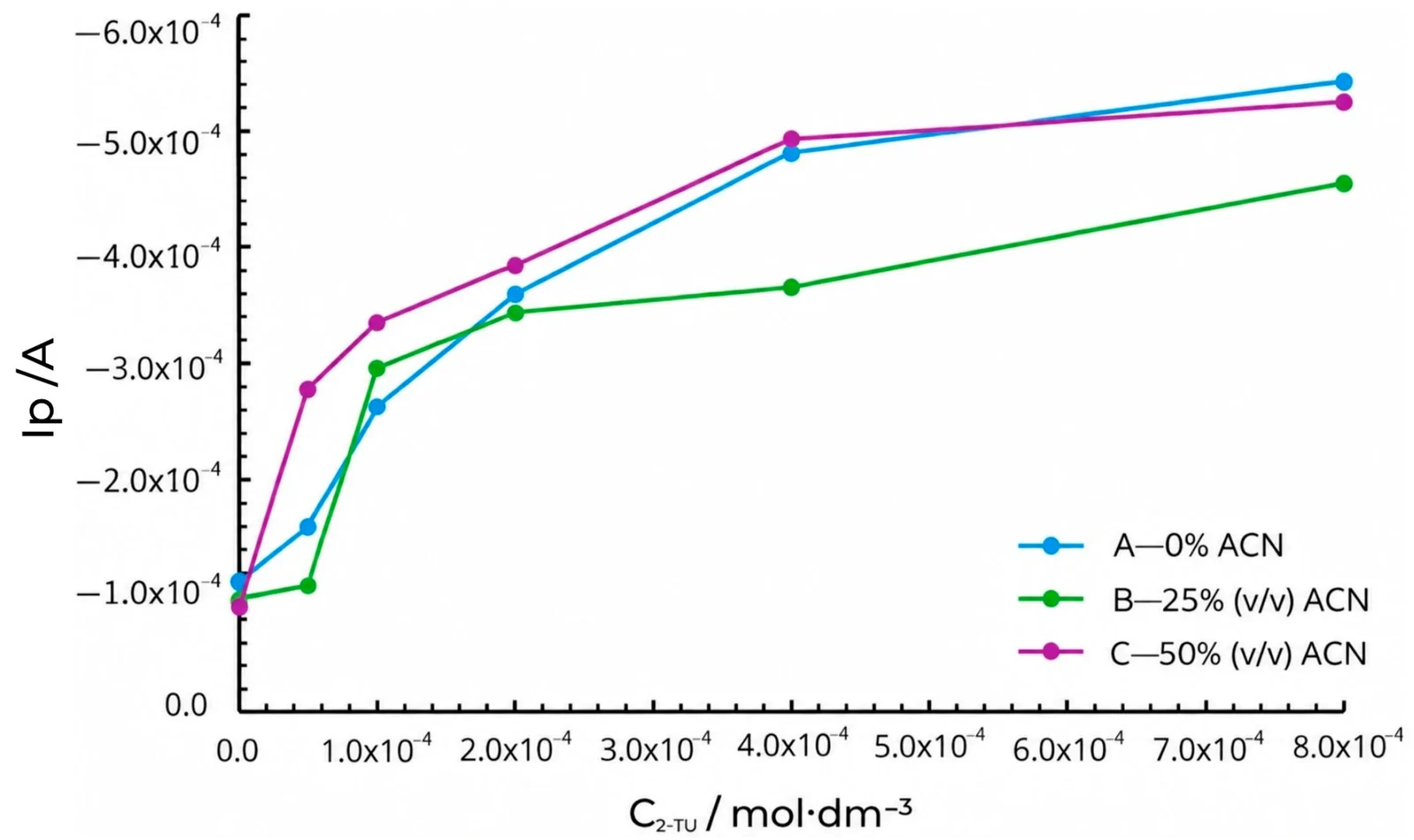
Dependence of the absolute SWV peak current |I_p_| of Bi(III) electroreduction on 2-TU concentration in solutions containing different ACN fractions: A, 0% ACN (blue); B, 25%_(v/v)_ ACN (green); and C, 50%_(v/v)_ ACN (violet). The corresponding complete sets of SWV curves are provided in the Supplementary Materials (Figures S5 and S6).

In all three media, the addition of 2-TU caused a marked increase in the absolute SWV peak current (Figure 7, Table 2). The accompanying peak potential changes are listed in Table 2 and can be inspected in the complete SWV curve sets provided in the Supplementary Materials (Figures S5 and S6). In solution A, the peak potential changed from −0.0389 V for the system without the additive to 0.015–0.021 V after addition of 2-TU, while |I_p_| increased from 112.23 to 542.03 μA. At the highest concentration, this corresponds to an almost fivefold increase relative to the base solution.

In solution B, after addition of 2-TU, the peak potential remained within 0.0339–0.0355 V, whereas |I_p_| increased from 97.75 to 455.42 μA, corresponding to a 4.66-fold increase relative to the solution without additive. In solution C, E_p_ changed from 0.0371 to 0.0445 V and |I_p_| increased from 90.18 to 525.12 μA, corresponding to a 5.82-fold increase. At low 2-TU concentrations, the strongest relative current enhancement was observed in 50%_(v/v)_ ACN, whereas at higher concentrations, the aqueous system reached a comparable response.

The dependence of peak current on 2-TU concentration differed between the three media. In solution A, the increase was gradual; in solution B, a pronounced rise was observed from 1 × 10^−4^ mol·dm^−3^ 2-TU, whereas in solution C, a strong increase occurred already at the lowest 2-TU concentration. Because the SWV peak current is influenced by several experimental and interfacial factors, these changes should not be interpreted directly as changes in electron transfer kinetics. This is also reflected by the incomplete correspondence between |I_p_| and k_s,app_, particularly in the mixed perchlorate/acetonitrile media.

Introducing 2-TU decreased ΔE_p_ (Table 3, Table 4 and Table 5), indicating an increase in the apparent reversibility of Bi(III) electroreduction. This trend was observed over the investigated scan rate range, although its magnitude depended on the supporting-electrolyte composition and 2-TU concentration. In all systems, ΔE_p_ increased with scan rate, consistent with quasi-reversible behaviour. The relatively small ΔE_p_ values at lower scan rates in the presence of 2-TU may also indicate coupling of electron transfer with a chemical or interfacial step. Considered together with the differential capacitance response and the absence of systematic changes in the formal potential the results are consistent with the possible formation of labile Bi(III)-2-TU-associated species in the near-electrode region that could facilitate the overall electroreduction process [5,6,36,37].

#### 3.2.3. Kinetic Parameters Determined from CV

To compare the effect of 2-TU on Bi(III) electroreduction quantitatively, the effective cathodic transfer coefficient α, the apparent diffusion coefficient of the oxidised form of the depolariser D_ox_, and the apparent heterogeneous electron transfer rate parameter k_s,app_ were determined. The values obtained for solutions A–C are summarised in Table 6.

The electrochemical parameters indicate that the effect of 2-TU depends on its concentration and the electrolyte composition. In solution A, the effective α increased from 0.33 to about 0.49, while in solution B, it rose from 0.28 to 0.47 after initially dropping to 0.24 at the lowest 2-TU level. These shifts were linked to increases in k_s,app_ from 1.66 × 10^−4^ to 5.64 × 10^−3^ cm·s^−1^ in solution A, and from 1.41 × 10^−4^ to 5.36 × 10^−3^ cm·s^−1^ in solution B. Together with the decrease in ΔE_p_ (Table 3, Table 4 and Table 5), these findings suggest that 2-TU enhances apparent reversibility and the apparent heterogeneous electron transfer rate in these media.

In solution C, a more complex relationship was observed. The effective α increased from 0.25 to 0.47 at 2 × 10^−4^ mol·dm^−3^ of 2-TU and then decreased to 0.40 at 8 × 10^−4^ mol·dm^−3^. Similarly, k_s,app_ rose from 1.14 × 10^−4^ cm·s^−1^ without 2-TU to 3.83 × 10^−3^ cm·s^−1^ at 4 × 10^−4^ mol·dm^−3^ but then dropped to 4.66 × 10^−4^ cm·s^−1^ at the highest concentration. This indicates that the acceleration effect of 2-TU is limited to a specific concentration range in the 50%_(v/v)_ ACN medium. At the highest additive levels, changes in the interfacial layer’s organisation or coverage may hinder favourable effects. This behaviour suggests competition between the formation of labile Bi(III)-2-TU species and partial blocking or reorganisation of the electrode interface. Dox levels increased with higher 2-TU concentrations, especially in ACN media, but in this multi-step system, these Dox values should be viewed comparatively rather than as direct measures of molecular diffusion.

The formal potential data add to the kinetic analysis (Table 7). E_f_^0^ showed no clear monotonic trend with 2-TU concentration: values ranged from 0.008 to 0.012 V in solution A and 0.019 to 0.028 V in solutions B and C. The lack of systematic shifts in E_f_^0^ does not support a progressive increase in stable Bi(III)–2-TU complexes. When combined with the reductions in ΔE_p_, changes in effective α and k_s,app_, and the differential capacitance results, the data support the possible involvement of labile Bi(III)-2-TU species near the electrode. This aligns with the previously proposed CAP–PAIR model for Bi(III) electroreduction involving adsorbing S/N-donor substances [5,6,36,37]. Such species may facilitate partial dehydration of Bi(III) and the initial electron-transfer step, though this remains a hypothesis. The current methods do not reveal the stoichiometry or structure of these species. Confirmatory studies would require complementary spectroelectrochemical or speciation analyses under actual aqueous ACN conditions.

## 4. Conclusions

The combined analysis of differential capacitance, DC, SWV, and CV results showed that Bi(III) electroreduction at R-AgLAFE depends on both bulk medium properties and the organisation of the near-electrode region. Increasing the ACN fraction was associated with decreases in the current response, Dox, the effective α, and k_s,app_, together with an increase in ΔE_p_, indicating decreased apparent reversibility and kinetic response. These changes likely reflect combined transport, solvation, interfacial, and potentially reference potential contributions.

The addition of 2-TU was associated with changes in the interfacial response and, in most investigated systems, with increased apparent reversibility and k_s,app_. The absence of systematic changes in the formal potential, together with changes in ΔE_p_, the effective α, k_s,app_, and the differential capacitance response, is consistent with the possible participation of labile Bi(III)–2-TU-associated species in the near-electrode region. At 50%_(v/v)_ ACN and the highest 2-TU concentration, the accelerating effect weakened, which may reflect reorganisation or excessive coverage of the interfacial layer. Future microscopic studies could complement the present electrochemical results by examining whether detectable changes in the morphology of the deposited bismuth phase accompany changes in interfacial organisation.

## Figures and Tables

**Table 1:** Values of the effective cathodic transfer coefficient α, the apparent diffusion coefficient of the oxidised form of the depolariser D_ox_, and the apparent heterogeneous electron transfer rate parameter k_s,app_ determined for Bi(III) electroreduction in solutions A–C.

Solution	ACN [%_(v/v)_]	α	D_ox_ [cm^2^·s^−1^]	k_s,app_ [cm·s^−1^]
A	0	0.33	1.71 × 10^−5^	1.66 × 10^−4^
B	25	0.28	7.74 × 10^−6^	1.41 × 10^−4^
C	50	0.25	6.02 × 10^−6^	1.14 × 10^−4^

**Table 2:** Effects of 2-TU concentration on the SWV peak current of Bi(III) electroreduction in solutions A–C.

System	c(2-TU) [mol·dm^−3^]	|I_p_|, A [μA]	|I_p_|, B [μA]	|I_p_|, C [μA]
EP	0	112.23	97.75	90.18
C1	5 × 10^−5^	159.02	109.25	278.48
C2	1 × 10^−4^	262.73	295.83	335.42
C3	2 × 10^−4^	359.45	343.83	383.60
C4	4 × 10^−4^	481.95	365.75	493.03
C5	8 × 10^−4^	542.03	455.42	525.12

**Table 3:** Difference between the anodic and cathodic peak potentials ΔE_p_ for Bi(III) electroreduction with increasing 2-TU concentration and different scan rates in system A (0% ACN).

Solution	c(2-TU) [mol·dm^−3^]	ΔE_p_ [V] at Scan Rate v [V·s^−1^]							
		0.005	0.01	0.02	0.05	0.1	0.2	0.5	1.0
0.5 mol·dm^−3^ NaClO_4_ + 0.5 mol·dm^−3^ HClO_4_ + 1.0 × 10^−3^ mol·dm^−3^ Bi(NO_3_)_3_·5H_2_O (A)									
A	0	0.054	0.069	0.084	0.096	0.107	0.122	0.141	0.165
AC1	5 × 10^−5^	0.0420	0.047	0.042	0.051	0.057	0.064	0.073	0.079
AC2	1 × 10^−4^	0.032	0.036	0.041	0.048	0.055	0.062	0.071	0.077
AC3	2 × 10^−4^	0.032	0.037	0.042	0.050	0.056	0.063	0.072	0.078
AC4	4 × 10^−4^	0.033	0.037	0.042	0.050	0.057	0.064	0.072	0.078
AC5	8 × 10^−4^	0.031	0.035	0.040	0.048	0.054	0.061	0.070	0.076

**Table 4:** Difference between the anodic and cathodic peak potentials ΔE_p_ for Bi(III) electroreduction with increasing 2-TU concentration and different scan rates in system B (25%_(v/v)_ ACN).

Solution	c(2-TU) [mol·dm^−3^]	ΔE_p_ [V] at Scan Rate v [V·s^−1^]							
		0.005	0.01	0.02	0.05	0.1	0.2	0.5	1.0
0.5 mol·dm^−3^ NaClO_4_ + 0.5 mol·dm^−3^ HClO_4_ + 1.0 × 10^−3^ mol·dm^−3^ Bi(NO_3_)_3_·5H_2_O + 25%_(v/v)_ ACN (B)									
B	0	0.068	0.073	0.086	0.097	0.117	0.134	0.144	0.172
BC1	5 × 10^−5^	0.053	0.059	0.055	0.065	0.071	0.077	0.084	0.088
BC2	1 × 10^−4^	0.041	0.047	0.054	0.063	0.069	0.076	0.083	0.087
BC3	2 × 10^−4^	0.040	0.046	0.052	0.061	0.068	0.074	0.082	0.086
BC4	4 × 10^−4^	0.039	0.044	0.050	0.059	0.066	0.073	0.080	0.085
BC5	8 × 10^−4^	0.036	0.041	0.047	0.056	0.063	0.070	0.078	0.083

**Table 5:** Difference between the anodic and cathodic peak potentials ΔE_p_ for Bi(III) electroreduction with increasing 2-TU concentration and different scan rates in system C (50%_(v/v)_ ACN).

Solution	c(2-TU) [mol·dm^−3^]	ΔE_p_ [V] at Scan Rate v [V·s^−1^]							
		0.005	0.01	0.02	0.05	0.1	0.2	0.5	1.0
0.5 mol·dm^−3^ NaClO_4_ + 0.5 mol·dm^−3^ HClO_4_ + 1.0 × 10^−3^ mol·dm^−3^ Bi(NO_3_)_3_·5H_2_O + 50%_(v/v)_ ACN (C)									
C	0	0.084	0.088	0.091	0.104	0.126	0.146	0.169	0.198
CC1	5 × 10^−5^	0.066	0.062	0.069	0.078	0.085	0.090	0.096	0.101
CC2	1 × 10^−4^	0.045	0.051	0.058	0.067	0.074	0.079	0.086	0.089
CC3	2 × 10^−4^	0.044	0.050	0.057	0.066	0.072	0.078	0.085	0.088
CC4	4 × 10^−4^	0.042	0.049	0.055	0.064	0.071	0.077	0.084	0.088
CC5	8 × 10^−4^	0.040	0.046	0.053	0.062	0.069	0.075	0.082	0.086

**Table 6:** Values of the effective cathodic transfer coefficient α, the apparent diffusion coefficient of the oxidised form of the depolariser D_ox_, and the apparent heterogeneous electron transfer rate parameter k_s,app_ determined for Bi(III) electroreduction with increasing 2-TU concentration in solutions A–C.

c(2-TU) [mol·dm^−3^]	Solution A (0% ACN)			Solution B (25% ACN)			Solution C (50% ACN)		
	α	D_ox_ [cm^2^·s^−1^]	k_s,app_ [cm·s^−1^]	α	D_ox_ [cm^2^·s^−1^]	k_s,app_ [cm·s^−1^]	α	D_ox_ [cm^2^·s^−1^]	k_s,app_ [cm·s^−1^]
0	0.33	1.71 × 10^−5^	1.66 × 10^−4^	0.28	7.74 × 10^−6^	1.41 × 10^−4^	0.25	6.02 × 10^−6^	1.14 × 10^−4^
5 × 10^−5^	0.34	1.18 × 10^−5^	2.41 × 10^−3^	0.24	3.98 × 10^−5^	1.54 × 10^−3^	0.32	4.33 × 10^−5^	7.20 × 10^−4^
1 × 10^−4^	0.46	1.33 × 10^−5^	3.14 × 10^−3^	0.36	4.66 × 10^−5^	2.50 × 10^−3^	0.35	5.53 × 10^−5^	2.06 × 10^−3^
2 × 10^−4^	0.49	1.88 × 10^−5^	4.38 × 10^−3^	0.42	4.90 × 10^−5^	3.81 × 10^−3^	0.47	5.87 × 10^−5^	2.98 × 10^−3^
4 × 10^−4^	0.48	2.38 × 10^−5^	4.91 × 10^−3^	0.47	5.02 × 10^−5^	4.51 × 10^−3^	0.43	6.16 × 10^−5^	3.83 × 10^−3^
8 × 10^−4^	0.49	2.56 × 10^−5^	5.64 × 10^−3^	0.47	5.14 × 10^−5^	5.36 × 10^−3^	0.40	6.65 × 10^−5^	4.66 × 10^−4^

**Table 7:** Formal potentials E_f_^0^ and half-wave potentials E_r_^1/2^ of the Bi(III) reduction process with increasing 2-TU concentration in solutions A–C.

c(2-TU) [mol·dm^−3^]	Solution A (0% ACN)		Solution B (25% ACN)		Solution C (50% ACN)	
	E_f_^0^ [V]	E_r_^1/2^ [V]	E_f_^0^ [V]	E_r_^1/2^ [V]	E_f_^0^ [V]	E_r_^1/2^ [V]
0	0.011	0.009	0.028	0.026	0.028	0.024
5 × 10^−5^	0.012	0.010	0.020	0.017	0.020	0.020
1 × 10^−4^	0.010	0.008	0.021	0.018	0.021	0.018
2 × 10^−4^	0.012	0.011	0.021	0.019	0.021	0.019
4 × 10^−4^	0.008	0.006	0.019	0.019	0.019	0.018
8 × 10^−4^	0.008	0.008	0.020	0.018	0.020	0.020

## Data Availability

The original contributions presented in this study are included in the article/Supplementary Material. Further inquiries can be directed to the corresponding author.

## Supplementary Materials

The following supporting information can be downloaded at: https://www.mdpi.com/article/10.3390/molecules31183260/s1, Supplementary Part S1. Detailed kinetic treatment and calculation procedure; Supplementary Part S2. Enlarged differential-capacitance figures from the main manuscript and supplementary charts; References. Figure S1: Enlarged version of Figure 1 from the main manuscript. Differential-capacitance curves for supporting electrolytes containing 0, 25, and 50%_(v/v)_ ACN (A–C); Figure S2: Enlarged version of Figure 2 from the main manuscript. Differential-capacitance curves for the chlorates(VII) supporting electrolyte (A) in the presence of increasing 2-TU concentrations (indicated in the legend); Figure S3. Enlarged version of Figure 3 from the main manuscript. Differential-capacitance curves recorded for solution B, containing 25%_(v/v)_ ACN, at increasing 2-TU concentrations (indicated in the legend); Figure S4. Enlarged version of Figure 4 from the main manuscript. Differential-capacitance curves recorded for solution C, containing 50%_(v/v)_ ACN, at increasing 2-TU concentrations (indicated in the legend); Figure S5. SWV curves of Bi(III) electroreduction in supporting-electrolyte solutions containing different ACN fractions: A, 0% ACN; B, 25%_(v/v)_ ACN; and C, 50%_(v/v)_ ACN. Supporting electrolyte: 0.5 mol·dm^−3^ NaClO_4_; + 0.5 mol·dm^−3^ HClO_4_; c(Bi(III)) = 1; Figure S6. SWV curves of Bi(III) electroreduction recorded at increasing 2-thiouracil concentrations in solutions containing: (a) 0% ACN, (b) 25%_(v/v)_ ACN, and (c) 50%_(v/v)_ ACN. Supporting electrolyte: 0.5 mol·dm^−3^ NaClO_4_ + 0.5 mol·dm^−3^ HClO_4_; c(Bi(III)) = 1.0 × 10^−3^ mol·dm^−3^ [16,17,18,19,20,21,22].
