# Supplementary material for: Kinetic and Interfacial Aspects of Bi(III) Ion Electroreduction in the Presence of Acetonitrile and 2-Thiouracil
Source: Molecules. 2026 Sep 14;31(18):3260. doi: 10.3390/molecules31183260 (PMC13610010; doi:10.3390/molecules31183260)
Supplement: Supplementary file 1 [file molecules-31-03260-s001.zip › molecules-4517951-supplementary.pdf]

molecules

MDPI

Supporting Information

Kinetic and Interfacial Aspects of Bi(III) Ion Electroreduction in the Presence of Acetonitrile and 2-Thiouracil

Alicja Natalia Pawlak [1], Agnieszka Nosal-Wiercińska [1,*], Robert Pietrzak [2], Sebastian Grzyb [3] and Selehattin Yilmaz [4]

1 Department of Analytical Chemistry, Institute of Chemical Sciences, Faculty of Chemistry, Maria Curie-Skłodowska University, Maria Curie-Sklodowska Sq. 3, 20-031 Lublin, Poland; alicja.pawlak@mail.umcs.pl
2 Faculty of Chemistry, Adam Mickiewicz University in Poznań, Uniwersytetu Poznańskiego 8, 61-614 Poznań, Poland; pietrob@amu.pl
3 Warsaw Medical and Technical Academy of Applied Sciences, Bitwy Warszawskiej 1920 r. 18, 02-366 Warsaw, Poland; sebastian.grzyb@wamt.edu.pl
4 Department of Chemistry-Analytical Chemistry, Faculty of Science, Çanakkale Onsekiz Mart University, 17100 Çanakkale, Turkey; seletyilmaz@hotmail.com
* Correspondence: agnieszka.nosal-wiercinska@mail.umcs.pl

Contents. Supplementary Part S1. Detailed kinetic treatment and calculation procedure; **Supplementary Part 2.** Enlarged differential-capacitance figures from the main manuscript and supplementary charts; References.

Supplementary Part S1. Detailed kinetic treatment and calculation procedure

This section provides the detailed calculation procedure used for the comparative kinetic analysis. The equations are retained from the applied electrochemical treatment. The terminology is aligned with the revised interpretation of the multistep Bi(III) process: α is treated as an effective cathodic transfer coefficient, D_{ox} as an apparent comparative diffusion parameter, and $k_{s,app}$ as an apparent heterogeneous electron-transfer rate parameter.

1. Parameters read directly from CV curves

For each CV curve, the cathodic peak potential (E_{pc}), anodic peak potential (E_{pa}), cathodic peak current (I_{pc}),), and anodic peak current (I_{pa}) were determined. The peak-potential separation was calculated according to the relationship:

$$\Delta E_p = E_{pa} - E_{pc} \tag{S1}$$

An increase in ΔE_p as a function of the concentration of the investigated substance was interpreted as a decrease in the kinetic reversibility of the process, with the reservation that, in the case of the multistep electroreduction of Bi(III), peak positions may also be affected by adsorption, changes in the ionic environment in solution, mass transport, and interfacial properties.

The formal potential E_f^0 was determined from the characteristic potentials corresponding to one-half and one-quarter of the limiting current, read for the cathodic and reverse branches of the curve. The value of the cathodic transfer coefficient α was also included in the calculations, according to the equation [S1,S5]:

$$E_f^0 = \frac{1}{2}\left(E_{\frac{1}{2}}^r + E_{\frac{1}{2}}^k + \frac{\left(E_{\frac{1}{2}}^r + E_{\frac{1}{2}}^k\right) - \left(E_{\frac{1}{4}}^r + E_{\frac{1}{4}}^k\right)}{\alpha - 1}\right) \tag{S2}$$

https://doi.org/10.3390/molecules31183260

As a comparative parameter, $E^r_{\frac{1}{2}}$ the reversible half-wave potential was determined from the positions of the anodic and cathodic peaks [S1,S2,S4,S5]:

$$E^r_{\frac{1}{2}} = \frac{E_{pa}+E_{pc}}{2} \quad \text{(S3)}$$

The diffusion coefficient (D_{ox})of Bi(III) ions (was determined by a comparative method from direct-current polarographic curves (DCP) using a Bi(III) solution with a known diffusion coefficient. Based on the reference-solution measurement, the electrode calibration parameter h_{ref} was determined. The diffusion coefficient in the investigated systems was calculated from the equation [S1]:

$$D_{ox} = \left(\frac{I_{\frac{1}{2}} \cdot \sqrt{D_{ref}}}{3h_{ref}}\right)^2 \quad \text{(S4)}$$

where $I_{\frac{1}{2}}$ denotes the Bi(III) electroreduction current read from the DC curve at the half-wave potential, D_{ref} is the diffusion coefficient of Bi(III) ions, and h_{ref} is the calibration coefficient determined for the reference system [S1], whereas the number 3 corresponds to the number of electrons involved in the overall reduction of Bi(III) to Bi(0).

The product of the cathodic transfer coefficient and the number of electrons involved in the electrode process, αn_α, was determined from cyclic-voltammetry (CV) curves on the basis of the difference between the cathodic peak potential E_{pc} and the potential on the rising branch of the peak corresponding to half of its height, $E_{\frac{p}{2}}$. For a process treated approximately as completely irreversible, the Nicholson–Shain relationship was used [S3]:

$$\alpha n_a = \frac{1{,}857RT}{F|E_{pc}-E_{p/2}|} \quad \text{(S5).}$$

At 298.15 K, the equation took the form:

$$\alpha n_a = \frac{0{,}0477}{|E_{pc}-E_{p/2}|} \quad \text{(S6).}$$

Assuming formally the three-electron nature of the overall Bi(III) electroreduction, the effective value of the transfer coefficient was calculated as $\alpha_{eff} = \frac{\alpha n_\alpha}{3}$. This value was determined separately for each scan rate, and the mean over the range of scan rates at which the parameter reached relative stabilization was adopted as representative. Points corresponding to distorted peaks or clearly deviating from this range were omitted.

The apparent heterogeneous electron-transfer rate parameter ($k_{s,app}$) was determined by the Nicholson method from the peak-potential separation (ΔE_p). For each value, the dimensionless Nicholson kinetic parameter (Ψ) was calculated directly from the approximation equations of Lavagnini, Antiochia, and Magno. In the first step, the following value was determined:

$$X = n\Delta E_p \cdot 1000 \qquad \text{z} \qquad \text{(S7)}$$

where ΔE_p was expressed in volts, whereas X was expressed in millivolts. For Equation (9) of Lavagnini et al. [S6], the following was used:

$$\Psi = \frac{-0{,}6288+0{,}0021X}{1-0{,}017X} \quad \text{(S8)}$$

Equation (10), based on the Klingler–Kochi relationship and reported by Klingler and Kochi [S7] and incorporated into the extended procedure of Lavagnini et al. [S6], was used:

$$\Psi = 2{,}18\sqrt{\frac{\beta}{\pi}}\exp\left(-\frac{\beta^2 nF\Delta E_p}{RT}\right) \quad \text{(S9)}$$

The values were then calculated from the full Nicholson equation [S2]:

https://doi.org/10.3390/molecules31183260

$$k_{s,app} = \Psi \left(\frac{D_{\mathrm{red}}}{D_{\mathrm{ox}}}\right)^{\alpha/2} \sqrt{\frac{\pi n F v D_{\mathrm{ox}}}{RT}} \tag{S10}$$

where denotes the dimensionless Nicholson kinetic parameter; β is the transfer coefficient used in the Klingler–Kochi equation; D_{ox} and D_{red} are the diffusion coefficients of the oxidized and reduced forms of the depolarizer $[cm^2 \cdot s^{-1}]$, respectively; α is the cathodic transfer coefficient; n is the number of electrons; F is the Faraday constant $[C \cdot mol^{-1}]$; v is the scan rate; R is the gas constant; and T is the temperature.

2. Interpretative scope. Because Bi(III) electroreduction is a multistep process and may be coupled to adsorption, solvation, and interfacial reorganisation, the transfer coefficient is treated as an effective parameter and the heterogeneous kinetic quantity is reported as an apparent comparative parameter, $k_{s,app}$, rather than as an elementary standard rate constant for a single electron-transfer step. The value n = 3 represents the formal overall stoichiometry of Bi(III) reduction to Bi(0) and must not be interpreted as a single elementary three-electron-transfer step.

https://doi.org/10.3390/molecules31183260

Supplementary Part S2. Enlarged differential-capacitance figures from the main manuscript and supplementary charts

Figures S1-S4 reproduce Figures 1-4 of the main manuscript at a larger size to improve legibility of the numerous differential-capacitance curves.

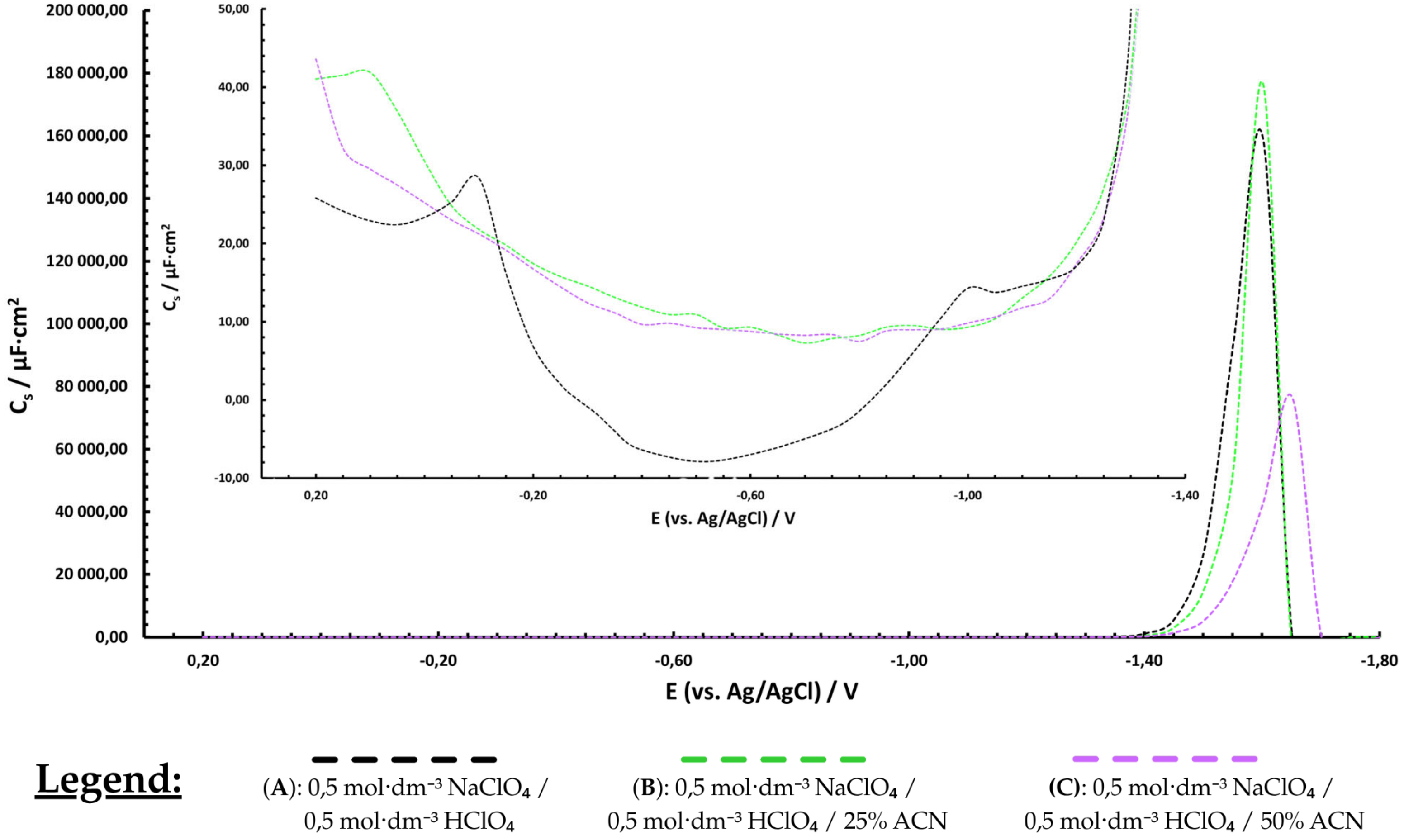

Figure S1. Enlarged version of Figure 1 from the main manuscript. Differential-capacitance curves for supporting electrolytes containing 0, 25, and 50%$_{(v/v)}$ ACN (A-C).

https://doi.org/10.3390/molecules31183260

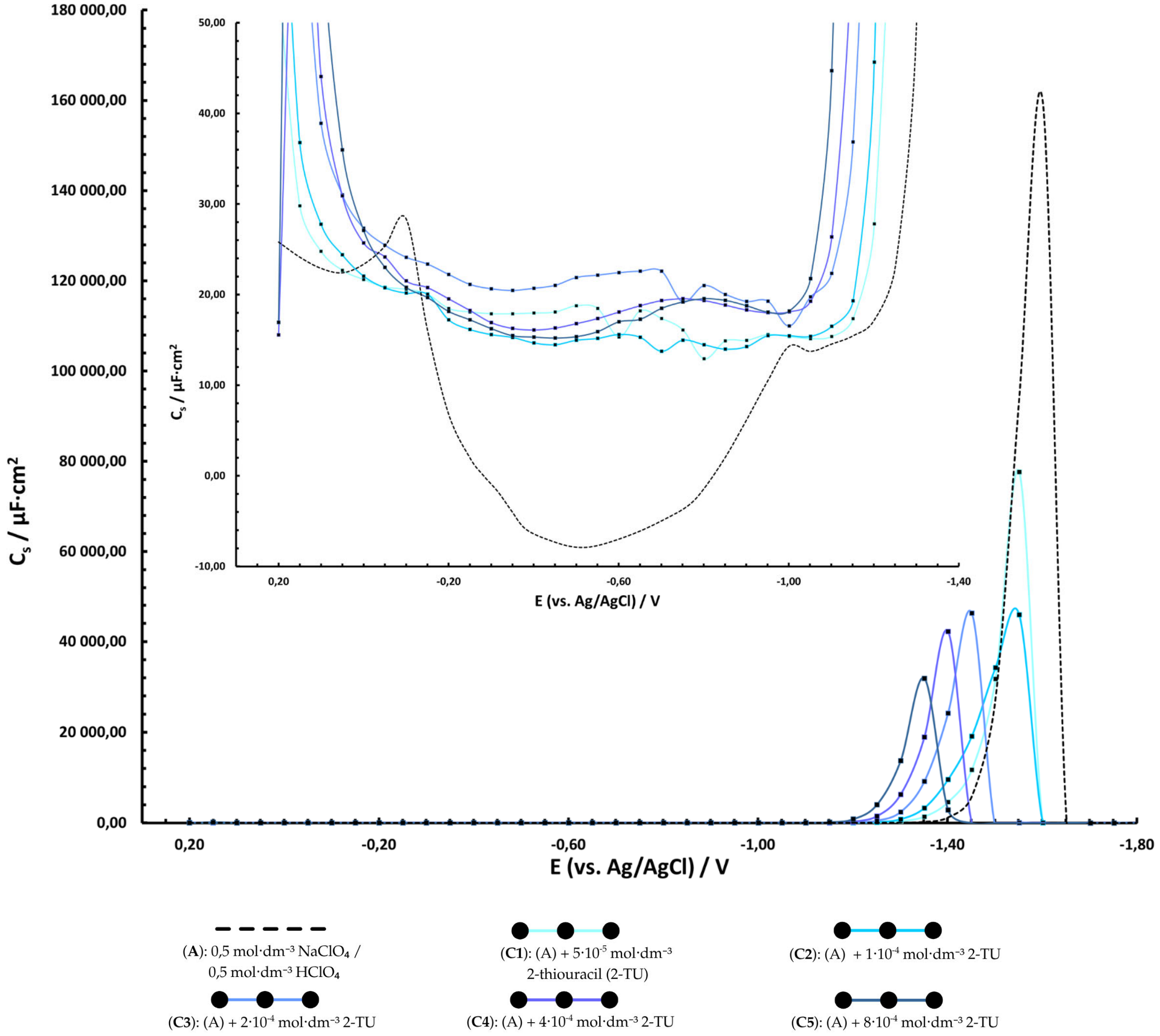

Figure S2. Enlarged version of Figure 2 from the main manuscript.

Differential-capacitance curves for the chlorates(VII) supporting electrolyte (A) in the presence of increasing 2-TU concentrations (indicated in the legend).

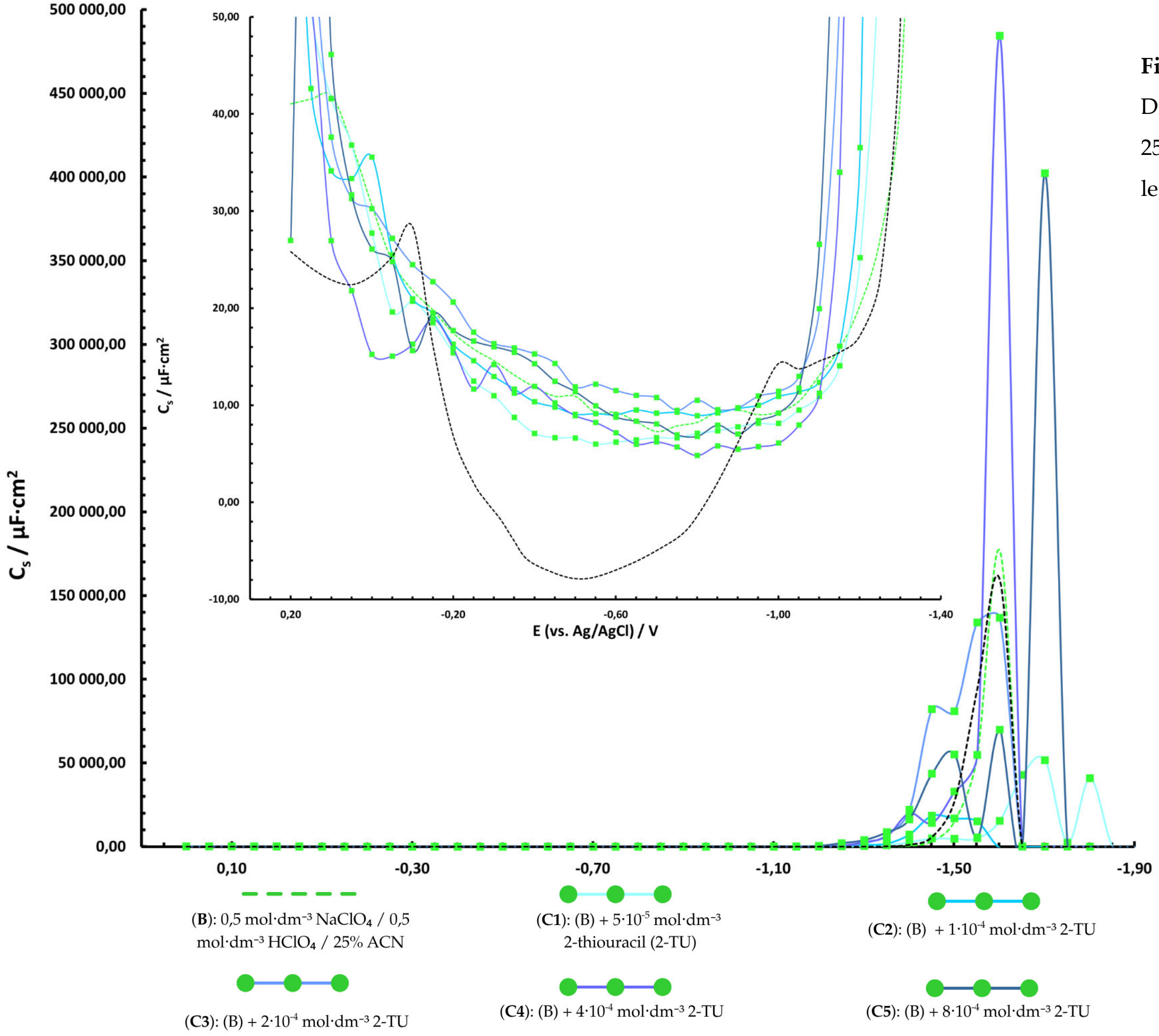

Figure S3. Enlarged version of Figure 3 from the main manuscript. Differential-capacitance curves recorded for solution B, containing 25%$_{(v/v)}$ ACN, at increasing 2-TU concentrations (indicated in the legend).

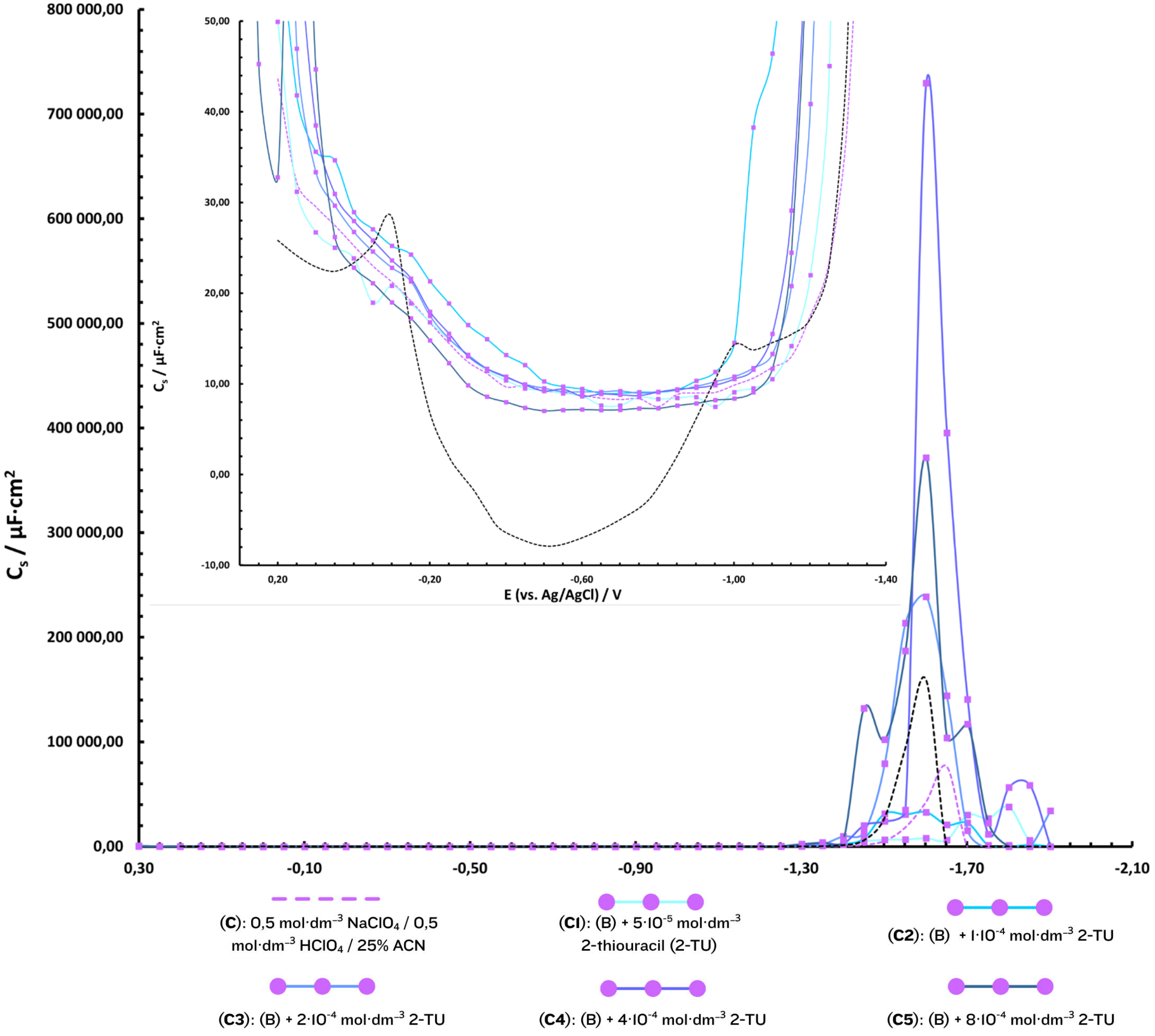

Figure S4. Enlarged version of Figure 4 from the main manuscript. Differential-capacitance curves recorded for solution C, containing 50%(v/v) ACN, at increasing 2-TU concentrations (indicated in the legend).

https://doi.org/10.3390/molecules31183260

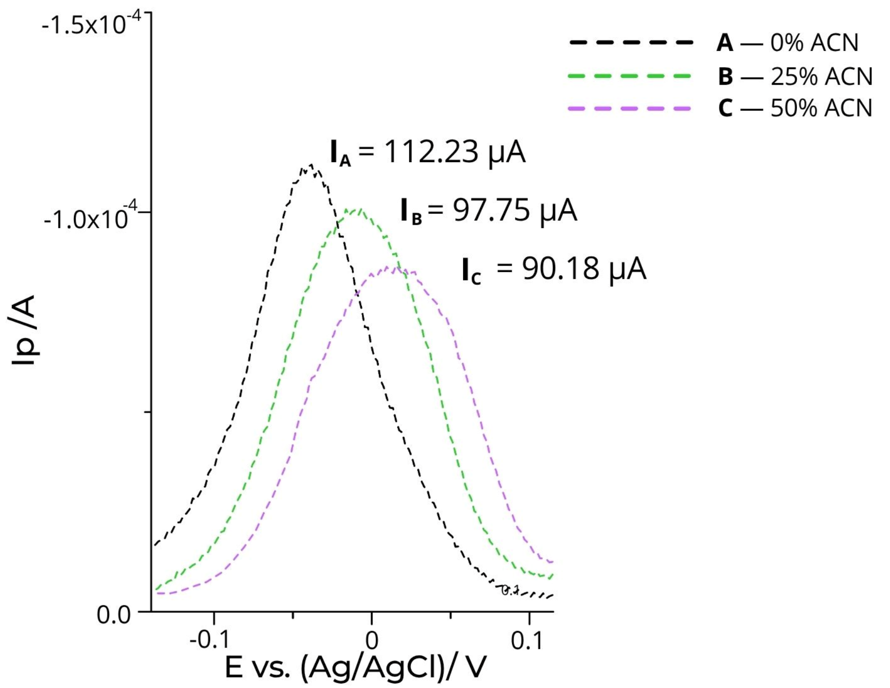

Figure S5. SWV curves of Bi(III) electroreduction in supporting-electrolyte solutions containing different ACN fractions: A, 0% ACN; B, 25% (v/v) ACN; and C, 50% (v/v) ACN. Supporting electrolyte: 0.5 mol·dm^{-3} $NaClO_4$ + 0.5 mol·dm^{-3} $HClO_4$; c(Bi(III)) = 1

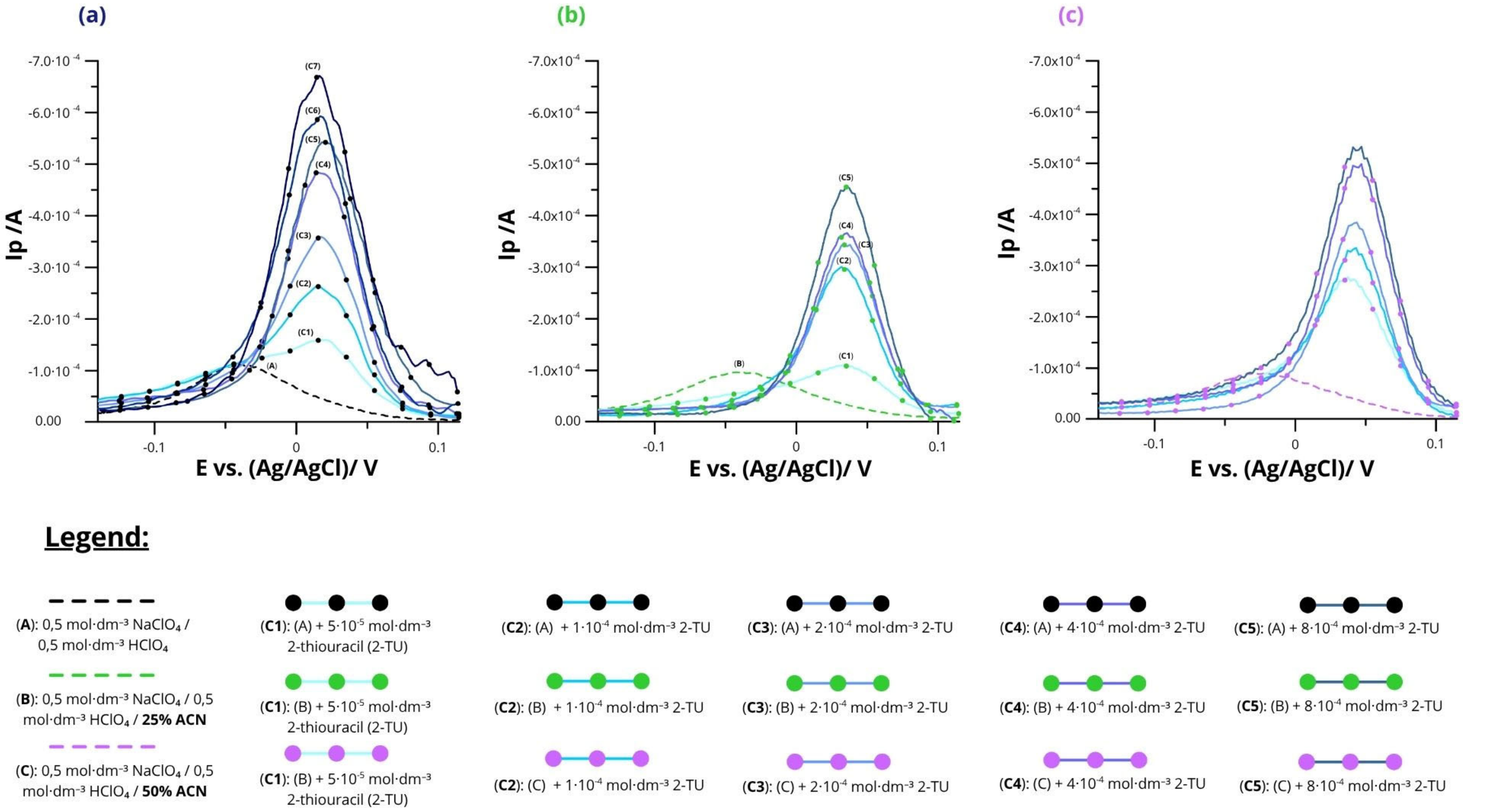

Figure S6. SWV curves of Bi(III) electroreduction recorded at increasing 2-thiouracil concentrations in solutions containing: (a) 0% ACN, (b) 25% (v/v) ACN, and (c) 50% (v/v) ACN. Supporting electrolyte: 0.5 mol·dm^{-3} $NaClO_4$ + 0.5 mol·dm^{-3} $HClO_4$; c(Bi(III)) = 1.0 × 10^{-3} mol·dm^{-3}.

https://doi.org/10.3390/molecules31183260

References

16. Bard, A.J.; Faulkner, L.R.; White, H.S. Electrochemical Methods: Fundamentals and Applications, 3rd ed.; Wiley: Hoboken, NJ, USA, 2022.

17. Nicholson, R.S. Theory and application of cyclic voltammetry for measurement of electrode reaction kinetics. Anal. Chem. 1965, 37, 1351–1355. https://doi.org/10.1021/ac60230a016.

18. Nicholson, R.S.; Shain, I. Theory of stationary electrode polarography: Single scan and cyclic methods applied to reversible, irreversible, and kinetic systems. Anal. Chem. 1964, 36, 706–723. https://doi.org/10.1021/ac60210a007.

19. Pingarrón, J.M.; Labuda, J.; Barek, J.; Brett, C.M.A.; Camões, M.F.; Fojta, M.; Hibbert, D.B. Terminology of electrochemical methods of analysis (IUPAC Recommendations 2019). Pure Appl. Chem. 2020, 92, 641–694. https://doi.org/10.1515/pac-2018-0109.

20. Oldham, K.B.; Myland, J.C. Extracting parameter values from quasireversible cyclic voltammograms. J. Solid State Electrochem. 2012, 16, 3691–3693. https://doi.org/10.1007/s10008-012-1885-2.

21. Lavagnini, I.; Antiochia, R.; Magno, F. An extended method for the practical evaluation of the standard rate constant from cyclic voltammetric data. Electroanalysis 2004, 16, 505–506. https://doi.org/10.1002/elan.200302851.

22. Klingler, R.J.; Kochi, J.K. Electron-transfer kinetics from cyclic voltammetry: Quantitative description of electrochemical reversibility. J. Phys. Chem. 1981, 85, 1731–1741. https://doi.org/10.1021/j150612a028.

Disclaimer/Publisher's Note: The statements, opinions and data contained in all publications are solely those of the individual author(s) and contributor(s) and not of MDPI and/or the editor(s). MDPI and/or the editor(s) disclaim responsibility for any injury to people or property resulting from any ideas, methods, instructions or products referred to in the content.
